# The Highs and Lows of P Supply in Medical Cannabis: Effects on Cannabinoids, the Ionome, and Morpho-Physiology

**DOI:** 10.3389/fpls.2021.657323

**Published:** 2021-07-15

**Authors:** Sivan Shiponi, Nirit Bernstein

**Affiliations:** ^1^Institute of Soil Water and Environmental Sciences, Volcani Center, Rishon LeZion, Israel; ^2^The Robert H. Smith Faculty of Agriculture, Food and Environment, The Hebrew University of Jerusalem, Rehovot, Israel

**Keywords:** *Cannabis*, cannabinoids, development, efficiency, fertilization, nutrition, phosphorus, reproductive

## Abstract

Environmental conditions, including the availability of mineral nutrients, affect secondary metabolism in plants. Therefore, growing conditions have significant pharmaceutical and economic importance for *Cannabis sativa*. Phosphorous is an essential macronutrient that affects central biosynthesis pathways. In this study, we evaluated the hypothesis that P uptake, distribution and availability in the plant affect the biosynthesis of cannabinoids. Two genotypes of medical “drug-type” cannabis plants were grown under five P concentrations of 5, 15, 30, 60, and 90 mg L^–1^ (ppm) in controlled environmental conditions. The results reveal several dose-dependent effects of P nutrition on the cannabinoid profile of both genotypes, as well as on the ionome and plant functional physiology, thus supporting the hypothesis: (i) P concentrations ≤15 mg L^–1^ were insufficient to support optimal plant function and reduced photosynthesis, transpiration, stomatal conductance and growth; (ii) 30–90 mg L^–1^ P was within the optimal range for plant development and function, and 30 mg L^–1^ P was sufficient for producing 80% of the maximum yield; (iii) Ionome: about 80% of the plant P accumulated in the unfertilized inflorescences; (iv) Cannabinoids: P supply higher than 5 mg L^–1^ reduced Δ^9^-tetrahydrocannabinolic acid (THCA) and cannabidiolic acid (CBDA) concentrations in the inflorescences by up to 25%. Cannabinoid concentrations decreased linearly with increasing yield, consistent with a yield dilution effect, but the total cannabinoid content per plant increased with increasing P supply. These results reveal contrasting trends for effects of P supply on cannabinoid concentrations that were highest under <30 mg L^–1^ P, vs. inflorescence biomass that was highest under 30–90 mg L^–1^ P. Thus, the P regime should be adjusted to reflect production goals. The results demonstrate the potential of mineral nutrition to regulate cannabinoid metabolism and optimize pharmacological quality.

## Introduction

*Cannabis sativa* is receiving commercial and academic attention globally due to its therapeutic potential for modern medicine and increasing recreational use (Small, 2018). Recent changes in regulations drive a proliferation of research efforts toward understanding the plant’s medical effects (Malfait et al., 2000; Zuardi, 2006; Bonini et al., 2018). The increasing use of cannabis as a prescription drug makes understanding the effects of environmental factors and growing conditions on the plant and its chemical composition a high priority (Decorte and Potter, 2015; Saloner and Bernstein, 2021). More than 500 secondary metabolites have been identified in cannabis plants, including terpenoids, flavonoids, and cannabinoids, which are responsible for the therapeutic qualities (Chandra et al., 2017; Gonçalves et al., 2019; Milay et al., 2020).

Secondary metabolites are involved in the interaction of plants with their environment and survival functions, such as attracting pollinators, defense against herbivores and pathogens, plant competition, symbiosis, and responses to environmental stresses (Demain and Fang, 2000; Verpoorte et al., 2002). They have been harnessed for centuries by humanity for use as pharmaceuticals, food additives and flavors (Zhao et al., 2005). These compounds biosynthesis in the plant is regulated by genetic and environmental factors; therefore, elicitation has been used for directing excelled chemical quality (Gorelick and Bernstein, 2014).

Cannabinoids are secondary metabolites produced by cannabis and stored mainly in glandular trichomes on the plant’s inflorescences (Turner et al., 1978). More than 100 cannabinoids have been identified in cannabis (Berman et al., 2018; Gülck and Møller, 2020; Milay et al., 2020). The most abundant cannabinoids are the pentyl type Δ^9^-tetra-hydrocannabinol (THC), cannabidiol (CBD), cannabichromene (CBC), and cannabigerol (CBG) that are present in the plant mostly in their acidic form (THCA, CBDA, CBCA, and CBGA). The precursors for cannabinoid biosynthesis in the cannabis plant are derived from two pathways, the polyketide pathway and the deoxyxylulose phosphate/methyl-erythritol phosphate (DOXP/MEP) pathway (Flores-Sanches and Verpoorte, 2008). CBGA is the direct precursor for THCA, CBDA, and CBCA, and it originates from prenylation of geranyl diphosphate (GPP) to olivetolic acid (Gülck and Møller, 2020). Δ^9^-tetrahydrocannabivarin (THCV) and cannabidivarin (CBDV), propyl analogs of THC and CBD, are minor cannabinoids originating from GPP and divarinic acid that have shown important pharmacological activities (Russo, 2011; Bonini et al., 2018; Sarma et al., 2020).

The cannabinoid profile of the plant is dynamic, varies between plants and spatially within the plant (Bernstein et al., 2019a; Danziger and Bernstein, 2021a) and is affected by genetics (Turner et al., 1978; Clarke and Merlin, 2016) and growing conditions (Bernstein et al., 2019b; Danziger and Bernstein, 2021b). Environmental stresses have a potential to be used as management practices to elicit changes in the plant’s secondary metabolite profile (Gorelick and Bernstein, 2014, 2017). Abiotic factors such as drought (Caplan et al., 2019), growing media (Caplan et al., 2017), salinity (Yep et al., 2020), light spectrum (Magagnini et al., 2018; Danziger and Bernstein, 2021a), nutrient supply (Bernstein et al., 2019b; Saloner and Bernstein, 2021), and stress elicitors (Jalali et al., 2019) were found to induce changes in the cannabinoid profile of *Cannabis sativa* plants.

Nutrients are essential for major plant processes such as growth, source–sink relationships, respiration, photosynthesis, photooxidation and metabolites biosynthesis, and involve in regulation and signaling in the plant cell (Engels et al., 2012). Hence, understanding the plant mineral requirements is crucial for improving yield quantity and quality (Wiesler, 2012). Phosphorus is an essential macronutrient and a key element in nucleic acids and phospholipids, as well as in energy transfer processes in the cell. It therefore participates and affects central biosynthesis pathways (White and Hammond, 2008; Shen et al., 2011; Hawkesford et al., 2012). In *Arabidopsis* plants, P deprivation reduced the concentrations of 87 primary metabolites, altered the levels of 35 secondary metabolites, and increased most organic acids, amino acids and sugar levels (Pant et al., 2015).

Understanding effects of P on medical cannabis plants at the reproductive stage is important for regulation of the secondary metabolite profile in the plant material produced for the pharmacology industry. The hypothesis guiding the study was that P uptake into the plant and its distribution and availability in vegetative and reproductive organs, affect secondary metabolism in cannabis, which is accompanied by changes to the physiological state and the ionome. To test our hypothesis, we exposed the plants to five P treatments of 5, 15, 30, 60, and 90 mg L^–1^ (ppm) P at the reproductive stage of development, and tested plant development, physiology and chemical profiling of cannabinoids and minerals within the plant. The study was conducted comparatively with two medical cannabis cultivars differing in chemovar to assess genotypic sensitivity to P nutrition. The obtained results improve our understanding of cannabis plant science, and enable to direct optimization and standardization of the medical product for the benefit of those who need it.

## Materials and Methods

### Plant Material and Growing Conditions

Two commercial medicinal cannabis cultivars, “Royal Medic” (RM) and “Desert Queen” (DQ) (Teva Adir Ltd., Israel), representing two chemotypes—(i) high THC and low CBD (DQ) and (ii) balanced THC and CBD (RM)—were chosen for the study. To ensure uniformity between plants, the plants were vegetatively propagated from cuttings of the same mother plant. The rooted cuttings were cultivated under a long photoperiod of 18/6 (day/night) using metal halide bulbs. After 4 weeks, the rooted cuttings, selected for uniformity, were transplanted into 3-L pots in perlite 2 (1.2) in controlled-environment growing rooms for 10 additional days of vegetative growth under at 25°C and 60% air relative humidity. The plants of each cultivar were divided randomly into five treatment groups of six replicated plants each, and the plants were randomly arranged in the cultivation space. The plants in each group received one of five P concentrations (5, 15, 30, 60, and 90 mg L^–1^ P), and short photoperiod was applied (12/12-h light/dark) using high-pressure sodium bulbs (980 μmol m^–2^s^–1^, Greenlab by Hydrogarden, Petah Tikva, Israel) for 63 and 68 days for DQ and RM, respectively. This concentration range was chosen with the goal to target deficiency as well as over-supply of P, for identification of the optimal range of P supply for physiological performance, as well as P stress response on yield quantity and cannabinoid production. To ensure uniform growing conditions throughout the growing room, light intensity was measured weekly, at every 50 cm^2^, and adjusted so as not to exceed 10% variability. The spacing of the plants was 0.2 m^2^ plant^–1^, and the plants were arranged in the cultivation space such as to minimize overlapping between plants. A reflective aluminum material covered the growing room for maximum reflection and light uniformity. The nutrient solution contained (in mM): 10.42 N-NO${}_{3}^{-}$, 2.07 N-NH${}_{4}^{+}$, 2.56 K^+^, 2.99 Ca^+2^, 1.44 Mg^+2^, 1.47 S-SO${}_{4}^{-2}$, 0.06 Cl^–^, 0.021 Fe^+2^, 0.011 Mn^+2^, 0.009 B^+3^, 0.005 Zn^+2^, 0.0008 Cu^+2^, and 0.0003 Mo^+2^. Routine monitoring of the irrigation solution confirmed that the P concentration remained steady and in accord with the target concentrations; pH was kept at 5.5–6.0. The leachate solution volume was ∼30% and analyzed weekly for pH and P concentration (Supplementary Figure 3). P concentration in the leachate solution of the lowest P treatment was lower than in the fertigation solution, and under the highest P supply treatments it was higher than in the fertigation solution, and leachate pH was similar to the pH of the fertigation solution.

### Plant Architecture and Development

Biomass accumulation was measured at the experiment’s termination by destructive samplings. Fresh weight of vegetative organs (leaves, stem, and roots) and reproductive organs (inflorescence and inflorescence leaves) was measured immediately following dissection from the plants. Dry weight (DW) was measured after drying at 65°C for 72 h. Morphological parameters (plant height, stem diameter, and the number of nodes on the main stem) were measured once a week throughout the experiment. Stem diameter was measured with a digital caliper (YT-7201, Signet Tools International Co., Ltd., Shengang District, Taiwan) at the location 2 cm above the plant base. All measurements were conducted on six replicated plants from each treatment in each of the two cultivars tested.

### Gas Exchange Parameters (Photosynthesis, Stomatal Conductance, Transpiration Rate, and Intercellular CO_2_ Concentration)

A Licor 6400 XT system (LI-COR, Lincoln, NE, United States) was used for the measurements. The youngest mature leaf on the main stem was analyzed [CO_2_ concentration: 400 mg L^–1^, PPFD: 500 μmol (m^2^s) ^–1^]. The leaves’ temperature was kept at 25°C, and the relative humidity at 60%. The measurements were conducted twice during plant development, on day 26 and 54, on six replicated plants for each treatment in each cultivar.

### Photosynthetic Pigments

Five disks, 0.6 cm in diameter, were removed from the youngest mature fan leaf on the plant main stem after the leaf was washed twice in distilled water and blotted dry. The disks were placed in 0.8 ml 80% (v/v) ethanol and kept in −20°C until further analysis. For the extraction of the pigments from the tissue, the samples were heated to 95°C for 30 min, and the solution was collected. Then, 0.5 ml of 80% (v/v) ethanol was added to the remaining tissue, and the tubes were heated again for 30 min. The combined extract was mixed by vortex; 0.4 ml was diluted in 5 ml (v/v) acetone, and absorbance was measured at 663, 664, and 740 nm by Genesys 10 UV scanning spectrophotometer (Thermo Scientific, Waltham, MA, United States). Chlorophyll *a* and *b* and carotenoids were calculated according to Lichtenthaler and Welburn (1983).

### Inorganic Mineral Analysis

At the termination of the experiment, the plants were separated into leaves, stems, roots, inflorescences and inflorescence leaves (that were hand-trimmed from the inflorescences), weighed, rinsed in distilled water, and dried at 65°C for 72 h. When dried, the samples were weighed again for dry weight determination, ground, and acid-digested by two different procedures and analyzed for N, P, K, Ca, Mg, Fe, Zn, and Mn as described in Saloner et al. (2019). The biomass data, the concentration of P in the various plant organs and total P in the plant were used for the calculations of P proportion in specific plant organs (Eq. 1), P utilization efficiency (Eqs. 2, 3), P acquisition efficiency (Eq. 4), and yield efficiency (Eq. 5).

(1) $$P\,p\,o\,r\,p\,o\,r\,t\,i\,o\,n\,i\,n\,a\,n\,o\,r\,g\,a\,n=\frac{P\,i\,n\,t\,i\,s\,s\,u\,e\,\left(m\,g\right)}{P\,i\,n\,p\,l\,a\,n\,t\,\left(m\,g\right)}$$

(2) $$P\,U\,E_{t}=\frac{P\,l\,a\,n\,t\,d\,r\,y\,w\,e\,i\,g\,h\,t\,\left(g\right)}{P\,i\,n\,t\,h\,e\,p\,l\,a\,n\,t\,\left(m\,g\right)}$$

(3) $$P\,U\,E_{y}=\frac{I\,n\,f\,l\,o\,r\,e\,s\,c\,e\,n\,c\,e\,d\,r\,y\,w\,e\,i\,g\,h\,t\,\left(g\right)}{P\,i\,n\,t\,h\,e\,p\,l\,a\,n\,t\,\left(m\,g\right)}$$

(4) $$P\,A\,E=\frac{P\,i\,n\,t\,h\,e\,p\,l\,a\,n\,t\,\left(m\,g\right)}{R\,o\,o\,t\,d\,r\,y\,w\,e\,i\,g\,h\,t\,\left(g\right)}$$

(5) $$Yieldefficiency\left(\%\right)=\frac{I\,n\,f\,l\,o\,r\,e\,s\,c\,e\,n\,c\,e\,D\,W}{\begin{array}{c} T\,r\,e\,a\,t\,m\,e\,n\,t\,t\,h\,a\,t\,a\,c\,h\,i\,e\,v\,e\,d\,t\,h\,e\\ h\,i\,g\,h\,e\,s\,t\,i\,n\,f\,l\,o\,r\,e\,s\,e\,n\,c\,e\,D\,W \end{array}}*100$$

where P is phosphorus; PUEt is P utilization efficiency for total dry weight; PUE_*y*_ is P utilization efficiency for yield dry weight, and PAE is P acquisition efficiency.

### Cannabinoid Analysis

Cannabinoids were analyzed in inflorescences from two locations in each plant: the apical inflorescence of the main stem (primary inflorescence) and the apical inflorescence of the lowest branch of the main stem (secondary inflorescence). The inflorescences were sampled at the end of the experiment when ∼40% of the trichomes were of amber color. The sampled inflorescences were hand-trimmed and dried in the dark at 19.5°C and 45% air humidity for 2 weeks. The dried samples were crushed manually, 50 mg was placed in a glass vial, 10 ml 100% analytical ethanol was added, and the mixture was shaken for 1 h at room temperature. One milliliter of the extract was filtered through a polyvinylidene difluoride (PVDF) membrane filter of 0.22 μm pore size (Bar-Naor Ltd., Ramat Gan, Israel). The cannabinoid concentrations were analyzed using a high-performance liquid chromatography (HPLC) system (Jasco 2000 Plus series) in a spectrum mode. The system consisted of a quaternary pump, autosampler, column compartment, and photodiode array (PDA) detector (Jasco, Tokyo, Japan). Chromatographic separations were conducted with a Luna Omega 3 μm Polar C18 column (Phenomenex, Torrance, CA, United States), with a length of 150 mm and internal diameter of 2.1 mm, employing acetonitrile/water 75:25 (v/v) with 0.1% (v/v) formic acid, at the isocratic mode, with a flow rate of 1.0 ml/minute and run time of 20 min under 25°C. Quantification was based on analytical standards: CBC, CBD, CBDV, cannabichromenic acid (CBCA), cannabidiolic acid (CBDA), cannabidivarinic acid (CBDVA), Δ^9^-tetrahydrocannabivarinic acid (THCVA), cannabicyclol (CBL), cannabinol (CBN) from Sigma-Aldrich (Germany), cannabicitran (CBT) from Cayman Chemical (Ann Arbor, MI, United States), and THC, Δ^9^-tetrahydrocannabinolic acid [THCA (THCA-A)], and Δ^9^-tetrahydrocannabivarin (THCV) from Restek (Bellefonte, PA, United States). For all standards, the linear *R*^2^ of the calibration curves was >0.994 (Saloner and Bernstein, 2021). Concentrations of THCV, CBDV, CBL, CBN, and CBT were lower than the detection limits. The total amount of each cannabinoid in a plant (g/plant) was calculated as the average concentrations of the cannabinoid in the two inflorescences sampled, multiplied by the inflorescence DW yield per plant.

### Statistical Analysis

The data were statistically analyzed by two-way ANOVA, followed by a *post hoc* Tukey’s honest significant difference (HSD) test (α = 0.05). Comparison of relevant means was conducted using Fisher’s least significant difference test at 5% level of significance. Pearson correlation was calculated for cannabinoid concentration and yield production. The analysis was performed with the Jump software (Jump package, version 9, SAS 2015, Cary, NC, United States).

## Results

### Morphology and Biomass

**FIGURE 1:**
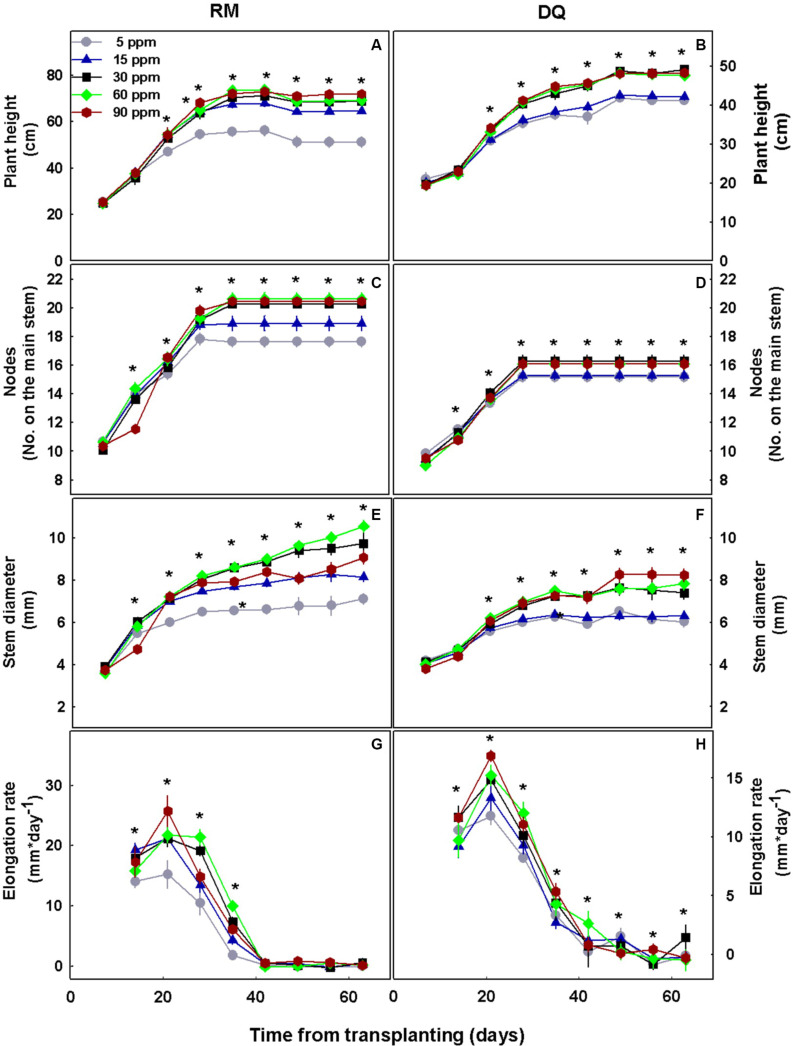
Effect of P concentration on development of two medical cannabis cultivars, RM and DQ, at the flowering phase. Plant height **(A,B)**, number of nodes on the main stem **(C,D)**, stem diameter **(E,F)**, and elongation rate **(G,H)**. The first measurement represents the time of initiation of the P treatments, and the short photoperiod. Presented data are averages ± SD (*n* = 6). An asterisk above the averages represents a significant difference between treatments for a given day by Tukey’s HSD test at α = 0.05.

**FIGURE 2:**
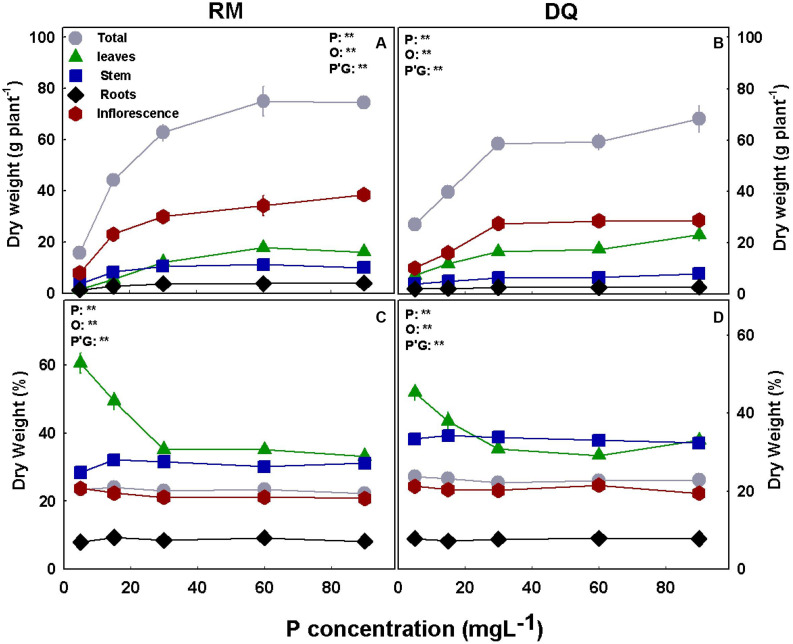
Effect of P nutrition on biomass of the root and shoot organs in mature medical cannabis plants. Dry weight **(A,B)** and percentage of dry weight **(C,D)** of leaves, stems, roots, inflorescences, and total plant of two medical cannabis cultivars, RM **(A,C)** and DQ **(B,D)**. Presented data are averages ± SD (*n* = 6). Results of two-way ANOVA indicated as **P < 0.05, *F*-test; NS, not significant *P* > 0.05, *F*-test. In the ANOVA results, P, phosphorus; G, genotype; and P*G, the interaction between P and G.

**FIGURE 3:**
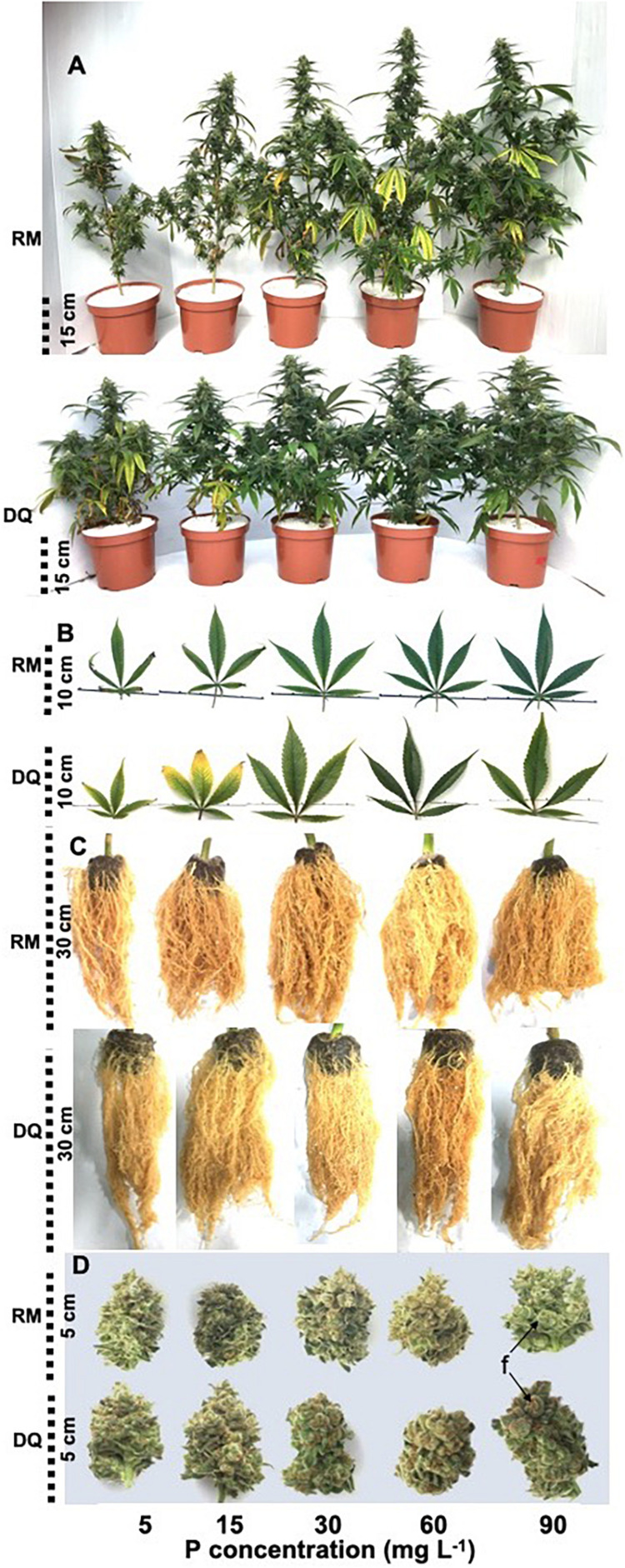
Effect of P supply on visual appearance of whole plants **(A)**, leaves **(B)**, roots **(C)**, and trimmed inflorescences **(D)** of two medical cannabis cultivars RM and DQ. The images were taken at plant maturity. Shown are the apical inflorescence on the main stem and the youngest fully developed leaf on the main stem. “f” points at individual flowers in the inflorescence.

P deficiency inhibited morphological development in both varieties as was apparent by the lower values of all morphological parameters tested under low P supply (Figure 1). DQ plants were more sensitive to low P concentration than RM, and growth restriction was apparent in DQ under 5 and 15 mg L^–1^ P, while in RM growth restriction was greater under 5 mg L^–1^ compared with 15 mg L^–1^ P. Phosphorous supply above 30 mg L^–1^ did not induce further growth stimulation. The elongation rate decreased from the third week of exposure to the short photoperiod and was lowest under 5 mg L^–1^ P in both genotypes. Biomass accumulation increased with P in both cultivars up to 30 mg L^–1^ P (Figure 2). Percent DW of the leaves was highest under P deficiency in both cultivars. Plants grown under P deficiency (5–15 mg L^–1^ P) were smaller than under higher supply rates, with fewer and chlorotic leaves. Furthermore, the inflorescences appeared less dense, and the individual flowers within the inflorescence appeared smaller (Figure 3).

### Gas Exchange and Pigments

**FIGURE 4:**
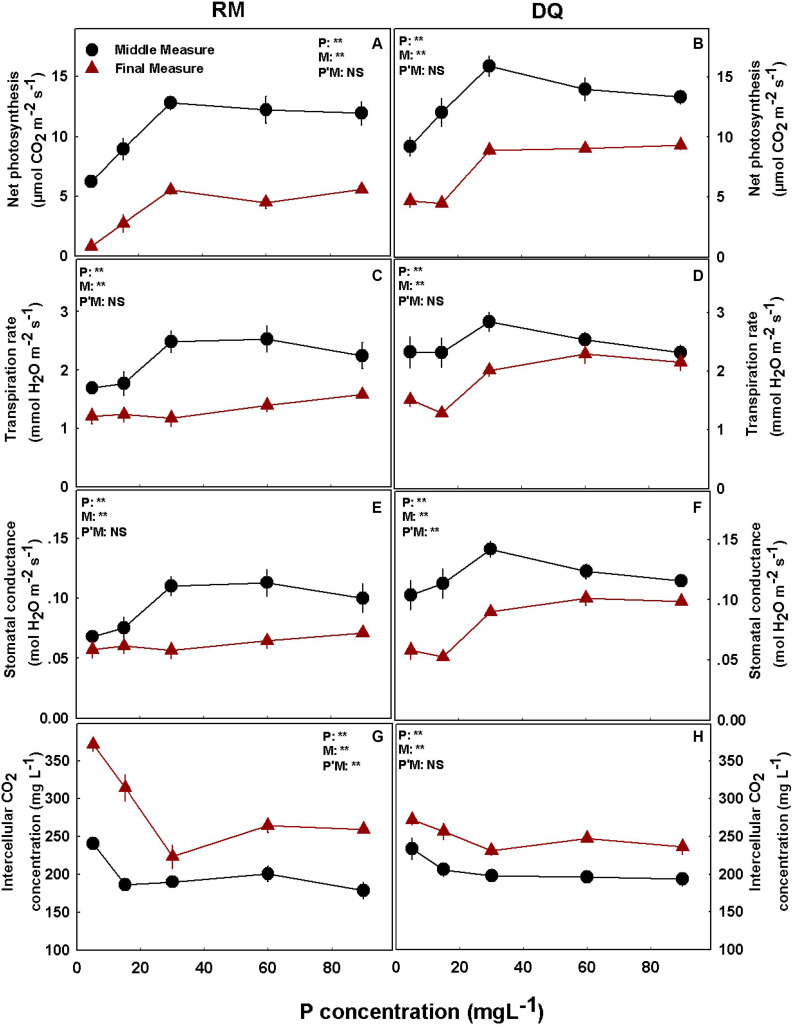
Effect of P supply on gas exchange parameters in cannabis leaves. Net photosynthesis rate **(A,B)**, transpiration rate **(C,D)**, stomatal conductance **(E,F)**, and intercellular CO_2_ **(G,H)** for two medical cannabis cultivars, RM and DQ. Results of measurements at two developmental stages, at the middle and at the end of the flowering phase. Presented data are averages ± SD (*n* = 6). Results of two-way ANOVA indicated as **P < 0.05, *F*-test; NS, not significant *P* > 0.05, *F*-test. In the ANOVA results, P*M, the interaction between P and measurement.

Under P deficiency (5 and 15 mg L^–1^ P), both cultivars had lower rates of photosynthesis, transpiration rate, and stomatal conductance and higher intercellular CO_2_ concentrations compared with higher supply rates (Figure 4). The measurements were conducted twice during plant development: at the middle and the end of the reproductive growth phase. At late maturation (second measurement), the plants were physiologically less active than earlier in development and had lower stomatal conductance, photosynthesis, and transpiration rates and higher intercellular CO_2_ (Figure 4). Photosynthesis was highest in both cultivars at the 30–90 mg L^–1^ P range, and a small decline above 30 mg L^–1^ P was found at the first measurement in DQ (Figures 4A,B). Transpiration rate and stomatal conductance were highest at the first measurement under 30–60 mg L^–1^ P in RM and 30 mg L^–1^ P in DQ. At the second measurement, transpiration rate and stomatal conductance were highest at 90 mg L^–1^ P in RM and 60–90 mg L^–1^ P in DQ (Figures 4C–F). Intercellular CO_2_ declined with the increase in P supply in both measurements; a small rise under 60 and 90 mg L^–1^ P was found at the second measurement of RM (Figures 4G,H). The photosynthetic pigments chlorophyll *a*, chlorophyll *b*, and carotenoids increased with the increase in P application up to 60 mg L^–1^ and did not change with further increase in P (Supplementary Figure 1).

### Nutrient Accumulation

**FIGURE 5:**
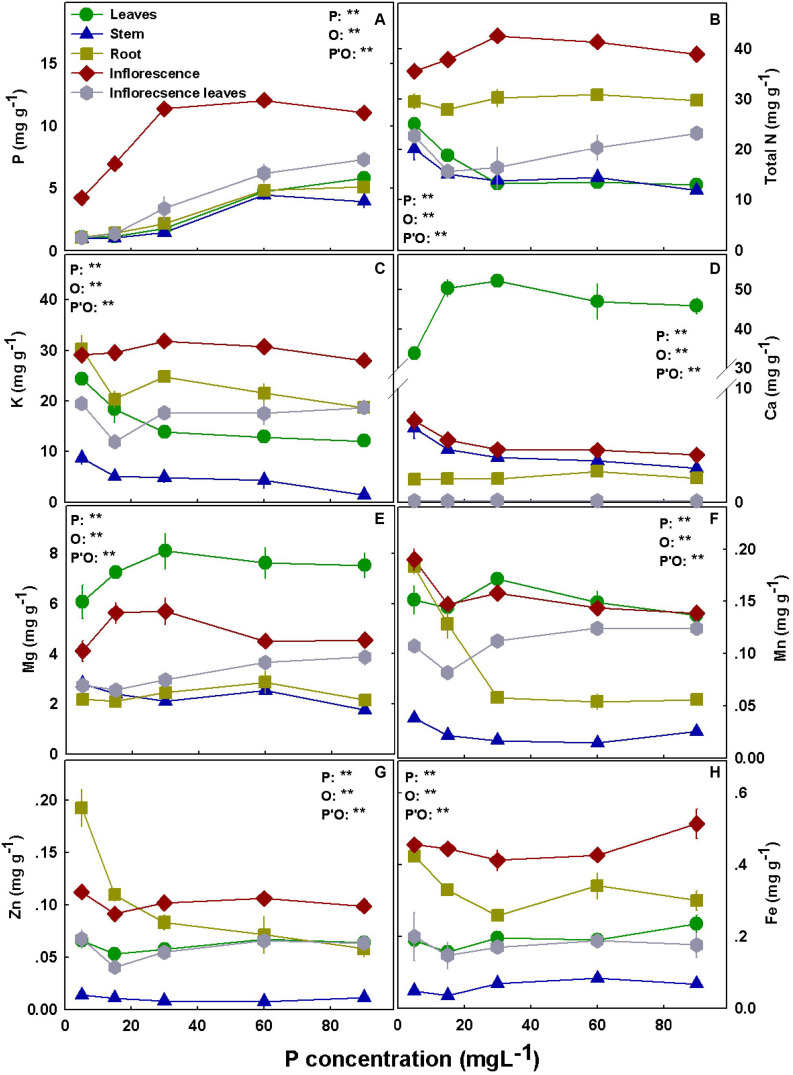
Effect of P supply on nutrient concentrations in leaves, stems, roots, and inflorescences in the medical cannabis cultivar RM. P **(A)**, N **(B)**, K **(C)**, Ca **(D)**, Mg **(E)**, Mn **(F)**, Zn **(G)**, and Fe **(H)**. The presented data are averages ± SD (*n* = 5); the concentrations are in mg g DW^–1^. Results of two-way ANOVA indicated as **P < 0.05, *F*-test; NS, not significant *P* > 0.05, *F*-test. In the ANOVA results, P, phosphorus; O, organ; and P*O, the interaction between P and O.

**FIGURE 6:**
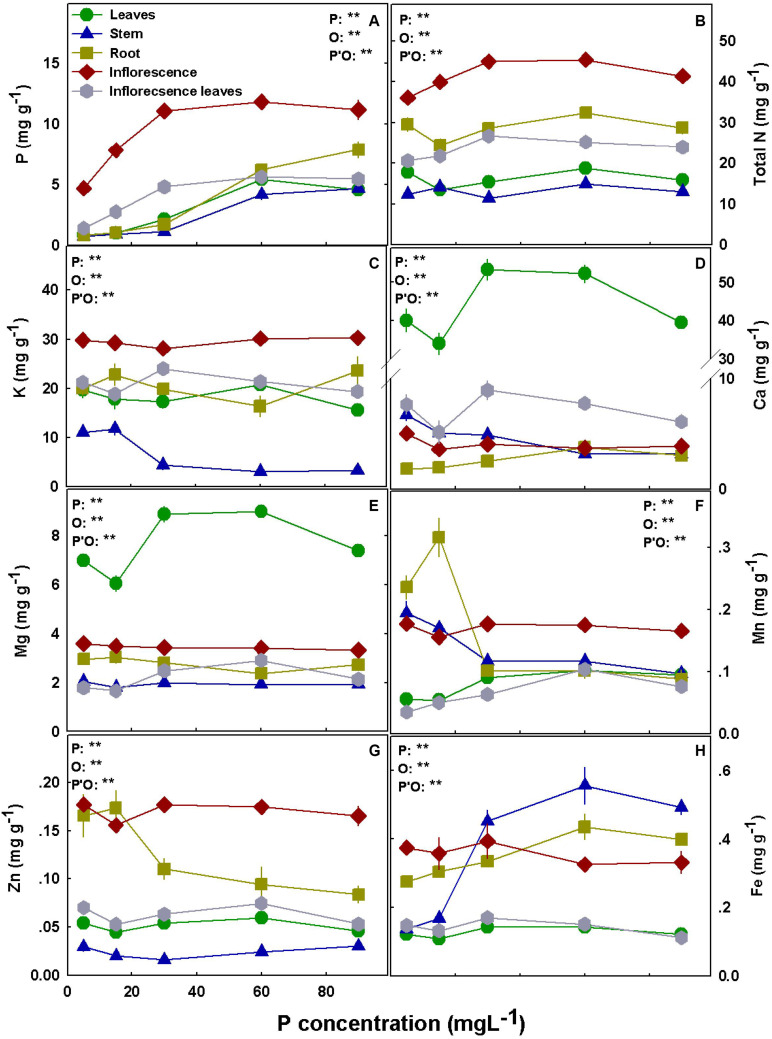
Effect of P supply on nutrient concentrations in leaves, stems, roots and inflorescences in the medical cannabis cultivar DQ. P **(A)**, N **(B)**, K **(C)**, Ca **(D)**, Mg **(E)**, Mn **(F)**, Zn **(G)**, and Fe **(H)**. The presented data are averages ± SD (*n* = 5); the concentrations are in mg g DW^–1^. Results of two-way ANOVA indicated as **P < 0.05, *F*-test; NS, not significant *P* > 0.05, *F*-test. In the ANOVA results, P, phosphorus; O, organ; and P*O, the interaction between P and O.

The distribution of the nutrients to the plant organs was nutrient specific. N, P, and K accumulated to the highest concentrations in the inflorescences. Ca and Mg concentrations were highest in the leaves in both cultivars, and high Mg accumulation was also found in RM inflorescences (Figures 5, 6).

**FIGURE 7:**
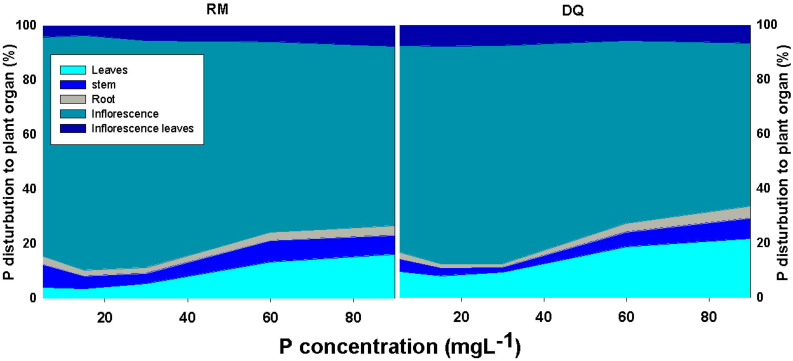
Effect of P supply on the distribution of P in the plant to leaves, stem, roots, inflorescences, and inflorescence-leaves, in two medical cannabis cultivars, RM and DQ. The total P content in each organ is presented as the percent content of the total P in the plant. Presented data are averages (*n* = 5).

Bioaccumulation of the minerals in the plant’s organs was affected by P supply (Figures 5, 6). P concentration in plant tissues increased with P supply in all plant organs up to 60 mg L^–1^ (Figures 5A, 6A). Interestingly, P accumulated in inflorescences to a higher proportion under P deficiency (Figure 7), and the relative accumulation in the vegetative organs compared to the reproductive tissue increased with the increase in P availability in the nutrient solution.

In both cultivars, N concentration in the inflorescences increased with the increase in P application up to 30 mg L^–1^ P (Figures 5B, 6B) but decreased with an increase in P up to 30 mg L^–1^ in RM’s leaves and stem. K concentrations in leaves, stem, and root of RM plants and DQ’s stem were highest under low P supply (Figures 5C, 6C). Ca concentration in the root increased with P supply in both cultivars, unlike the concentrations in the inflorescences and the stem that were highest under 5 mg L^–1^ P (Figures 5D, 6D). Leaves’ Ca reached a maximum concentration at 30–60 mg L^–1^ and decreased with further P supply. Like Ca, Mg concentration in DQ leaves also demonstrated a maximum response curve to P supply, while in the stem, roots, and inflorescence Mg concentrations were not affected by the level of P supplied (Figure 6F). However, Mg concentration in RM’s stem declined with the increase in P, Mg in the leaves increased up to 30 mg L^–1^ P, and Mg in the inflorescence increased as well up to 30 mg L^–1^ P but decreased with further increase in P availability.

Manganese concentration in the leaves presented a maximum response curve to P supply in RM and increased with increasing P up to 30 mg L^–1^ in DQ (Figures 5F, 6F). In the stem, Mn declined in both cultivars with the increase in P supply, and an increase in RM’s stem was found at 90 mg L^–1^. Mn in the inflorescences was not affected by the treatments in DQ and was highest under 5 mg L^–1^ P in RM, demonstrating a genotypic variability in response to P supply.

Zinc concentration was generally higher in roots and in the inflorescences compared with all other plant organs in both cultivars (Figure 5G, 6G). Zn retention in roots under P scarcity (5–15 mg L^–1^ P) was observed in both genotypes. Iron concentration in DQ’s leaves was not affected by the treatments and increased with P in RM (Figures 5H, 6H). In the stem, Fe concentration increased with increasing P supply up to 30 mg L^–1^ P in both cultivars and was higher in DQ. The root’s Fe response to the P treatments was genotype specific; it decreased with P in RM and increased in DQ. Inflorescence Fe was not affected significantly (*P* > 0.05) by the P treatments besides a slight increase at 90 mg L^–1^ P in RM.

### P Efficiency

**FIGURE 8:**
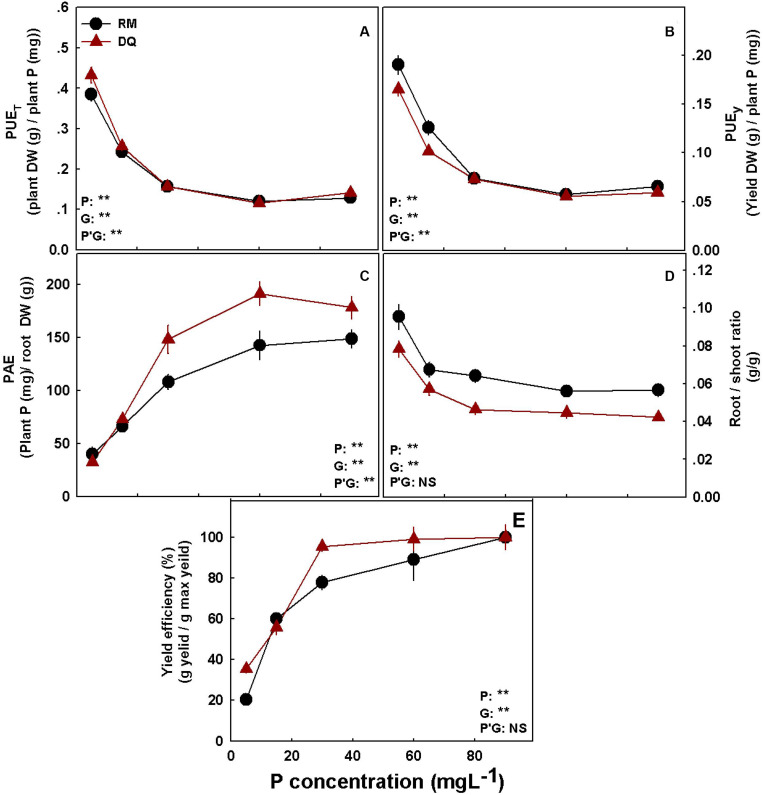
Phosphorus efficiency parameters for two medical cannabis cultivars RM **(A)** and DQ **(B)**. P utilization efficiency for total dry weight **(A)**, P utilization efficiency for yield dry weight **(B)**, phosphorus acquisition efficiency **(C)**, Root/shoot ratio **(D)**, and yield efficiency **(E)**. Presented data are averages ± SD (*n* = 5). Results of two-way ANOVA indicated as **P < 0.05, *F*-test; NS, not significant *P* > 0.05, *F*-test. In the ANOVA results, P*G, the interaction between P and genotype.

To assess P efficiency of the plant, five different parameters were calculated: (i) PUE_*t*_ (total DM/P), which analyzes the DM accumulation per P in the plant, (ii) PUE_*y*_ (flower DM/P), which analyzes the biomass of inflorescence produced per P in the plant, (iii) PAE (P/root), which calculates the P taken up by the plant per root unit, (iv) root/shoot ratio, and (v) yield efficiency (inflorescence DW/max inflorescence), which analyzes the percentage of yield in a defined treatment from the maximum yield achieved for the variety (Figure 8). The analyses revealed that PUE_*t*_ and PUE_*y*_ decline with the increase in P supply in both cultivars by up to 30 mg L^–1^ P and were not affected by a further increase in P supply (Figures 8A,B).

Phosphorus acquisition efficiency increased with the increase in P supply in both cultivars by up to 60 mg L^–1^ P (Figure 8C). DQ had a higher PAE value than RM. The root/shoot ratio decreased with P and was significantly higher for RM (Figure 8D). Both cultivars reached 80% of the maximum yield at 30 mg L^–1^ P (Figure 8E), yet DQ reached ∼20% higher yield under 30 mg L^–1^ P supply.

### Cannabinoids

**FIGURE 9:**
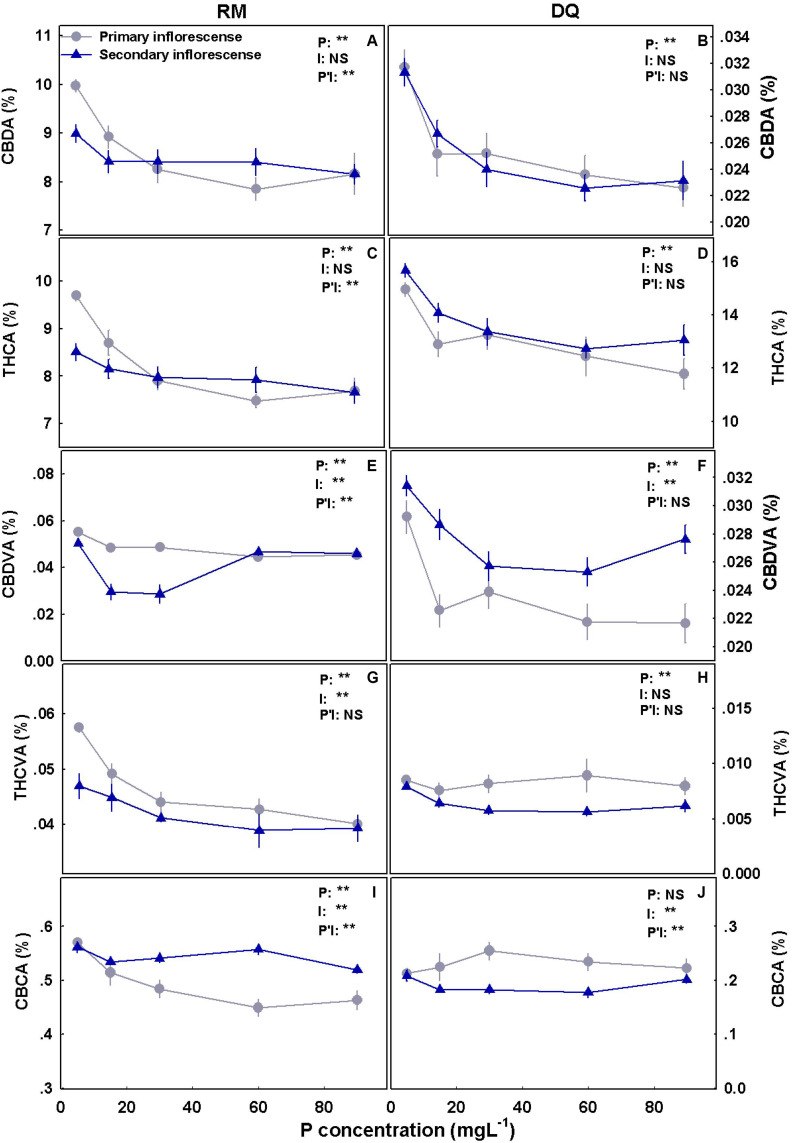
Effect of P application on cannabinoid concentrations in primary and secondary apical inflorescences in medical cannabis plants in two cultivars, RM and DQ. Cannabidiolic acid **(A,B)**, Δ^9^-tetrahydrocannabinolic acid **(C,D)**, cannabidivarinic acid **(E,F)**, Δ^9^-tetrahydrocannabivarinic acid **(G,H)**, and cannabichromenic acid **(I,J)**. The presented data are averages ± SD (*n* = 6). Results of two-way ANOVA indicated as **P < 0.05, *F*-test; NS, not significant *P* > 0.05, *F*-test. In the ANOVA results, P*I, the interaction between P and the inflorescence location.

Cannabinoid concentrations in the inflorescence were affected by the P treatments and overall reduced with the increase in P supply (Figure 9). THCA and CBDA concentrations had the most profound response to P concentrations and were reduced with P supplement in both genotypes and at both locations in the plant (i.e., in the primary and secondary inflorescences) (Figures 9A,B). Despite the considerable effect on THCA and CBDA, THC and CBD did not change significantly and were found at low concentrations (approximately 0.3 and 0.4% THC and 0.15% and undetected CBD in RM and DQ, respectively; Supplementary Figure 2). CBDVA was reduced with P in the primary inflorescence in both genotypes and demonstrated a minimum response curve in the secondary inflorescence (with a minimum at 15–30 and 30–60 mg L^–1^ in RM and DQ, respectively). THCVA concentration was reduced with P in DQ’s secondary inflorescences and in RM in both inflorescences. CBCA was reduced with P in RM’s upper inflorescence and was not affected in DQ. CBC concentration was low in both cultivars (RM: ∼0.03%, DQ: ∼0.003%) (Supplementary Figure 2).

**FIGURE 10:**
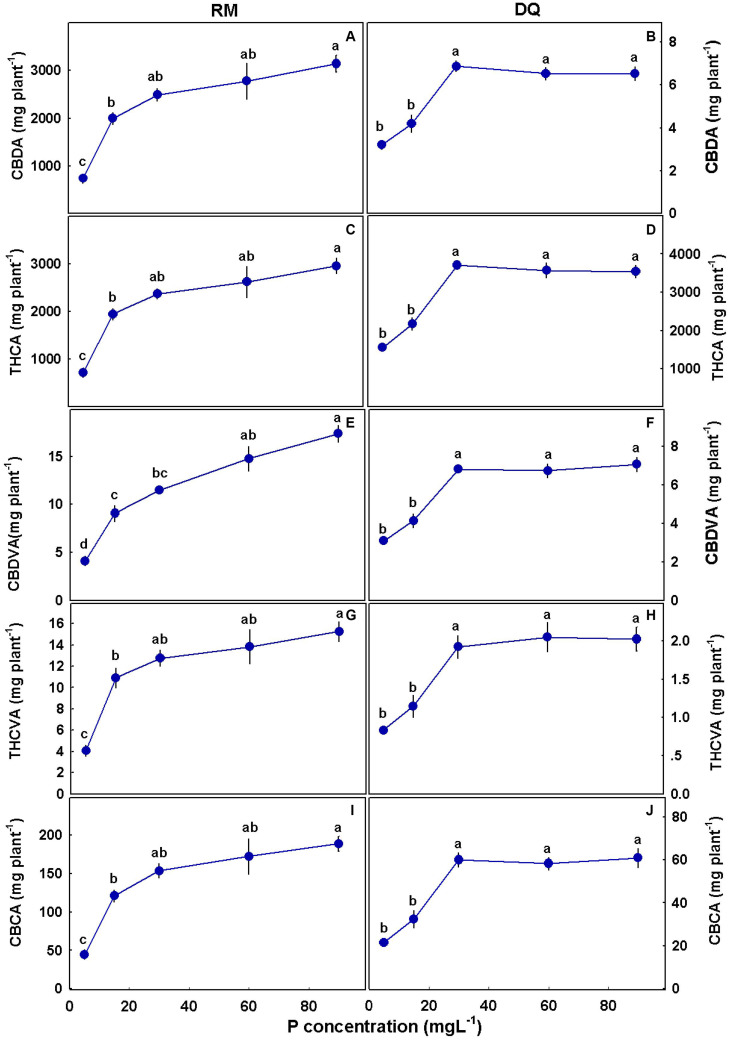
Effect of P application on cannabinoid yield per plant for two medical cannabis cultivars, RM and DQ. Cannabidiolic acid **(A,B)**, Δ^9^-tetrahydrocannabinolic acid **(C,D)**, cannabidivarinic acid **(E,F)**, Δ^9^-tetrahydrocannabivarinic acid **(G,H)**, and cannabichromenic acid **(I,J)**. The presented data are averages ± SD (*n* = 6). Different letters above the means represent significant differences according to Tukey’s honest significant difference test at α = 0.05.

The amount of cannabinoids produced per plant increased with P in RM for all cannabinoids tested (Figure 10). In DQ, such an increase was apparent only up to 30 mg L^–1^ P supply.

**FIGURE 11:**
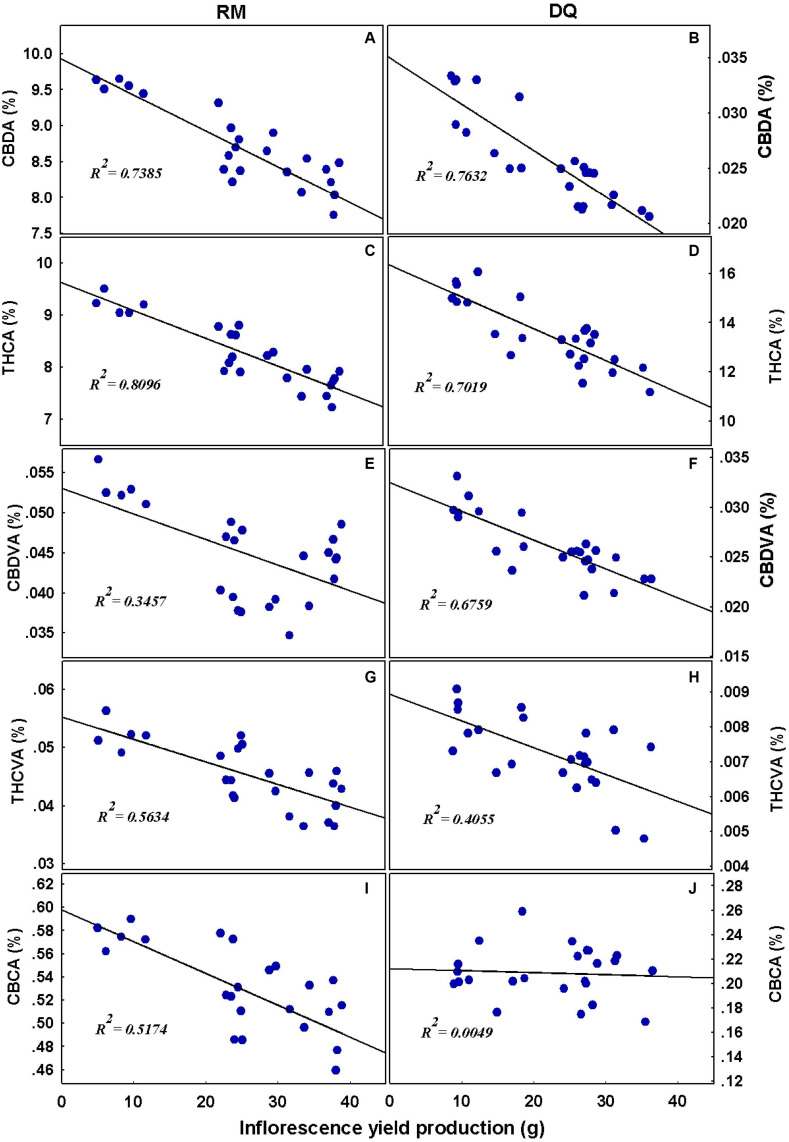
Linear regression analysis. Relationships between cannabinoid concentrations and inflorescence yield per plant. Cannabidiolic acid **(A,B)**, Δ^9^-tetrahydrocannabinolic acid **(C,D)**, cannabidivarinic acid **(E,F)**, Δ^9^-tetrahydrocannabivarinic acid **(G,H)**, and cannabichromenic acid **(I,J)**. The continuous line represents the linear fit to the data.

To understand the link between yield production and the cannabinoid concentrations, Pearson correlation coefficients were tested (Figure 11). In RM, CBDA, THCA, CBDVA, THCVA, and CBCA negatively correlated with yield; THCA and CBDA had the strongest negative correlation. The same results were obtained in DQ, except for CBCA which was not affected by yield in this genotype.

## Discussion

Phosphorus is a constituent of major compounds in the plant cells, such as nucleic acids and phospholipids, and it also plays a central role in energy transformations and as an energy carrier. It is therefore required for many key metabolic processes (Hawkesford et al., 2012). Thereby, the P status of the plant has a strong impact on plant development and metabolism (Wiesler, 2012). The present study evaluated effects of P supply on development and function of medical cannabis plants at the reproductive growth phase and on the profile of cannabinoids, the unique secondary metabolites in cannabis. The results reveal the importance of optimal P nutrition to the cannabis plant function and morpho-development as secondary metabolism was considerably affected by P supply as well as the plant gas exchange, CO_2_ fixation, mineral uptake and translocation, and P use efficiency. The foremost discovery is the contrasting effect of increasing P supply to increase inflorescence yield production but to decrease the biosynthesis of major cannabinoids, demonstrating that P supply needs to be regulated to suit yield quantity vs. chemical quality goals. The revealed influence of P on the cannabinoid profile can be utilized for adjusting the cannabidiome to achieve a desirable pharmacological profile in the product for medical purposes.

The sensitivity of plant growth and development to P supply at the reproductive phase presented in this study are similar to responses we have recently reported for the vegetative phase (Shiponi and Bernstein, 2021). Responses to N and K nutrition are described in Saloner et al. (2019) and Saloner and Bernstein (2020, 2021). In both phases of growth, morphological development and biomass deposition are inhibited under P starvation, and P concentrations up to 90 mg L^–1^ P do not result in toxicity. Additionally, in both phases of plant development, DQ plants are more sensitive to low P than RM plants, demonstrating similar genotypic sensitivity. Similar to our results, stunted growth under P deficiency was obtained also for hemp, and P addition above adequate supply did not affect the plant morphology and biomass (Vera et al., 2004, 2010). P toxicity is uncommon in plants because of the plant downregulation mechanisms of P uptake (Dong et al., 1999; Hawkesford et al., 2012).

The effect of P nutrition on leaf gas exchange parameters was likewise similar for the reproductive (Figure 4) and the vegetative stages (Shiponi and Bernstein, 2021) as well as for the middle and end of the reproductive phase. Photosynthesis, transpiration, and stomatal conductance were lowest under low P supply and reached a maximum under 30 mg L^–1^ P (Figure 4). Inhibition of photosynthesis under P deficiency was reported for many plants (Brooks, 1986; Wang et al., 2018; Taliman et al., 2019), and the growth restriction we identified under P deficiency could be a result of the lower photosynthesis rate. A decline in photosynthesis, transpiration rate, and stomatal conductance under higher P application (60 and 90 mg L^–1^) was found only in DQ plants at the first measurement. This resembles results that we have reported previously for the vegetative stage (Shiponi and Bernstein, 2021). Reduced photosynthesis as a result of P toxicity was observed in *Hakea prostrata* (Shane et al., 2004). However, the reduced photosynthesis under high P application did not affect DQ’s biomass or morphology, demonstrating that carbon fixation was not a limiting factor. P impact on photosynthesis rate was found to occur *via* two pathways: by effects on stomatal conductance or by a non-stomatal pathway involving enzymes of the Calvin cycle (Brooks, 1988; Fredeen et al., 1990; Wang et al., 2018). Due to the decrease in photosynthesis rate under low P, intercellular CO_2_ concentration increased in both cultivars and measurements, likely inducing the reduction in stomatal conductance (Allaway and Mansfield, 1967). The reduction in photosynthesis rate, together with the increase in intercellular CO_2_ concentration, suggests a non-stomatal restriction on CO_2_ assimilation under P deficiency. This result is unlike the response at the vegetative growth stage of medicinal cannabis, where a decrease in intercellular CO_2_ concentration was found under low P. The reduced chlorophyll concentration under P deficiency (Supplementary Figure 1) could have contributed to the observed restriction of photosynthetic activity. P starvation has been reported to decrease chlorophyll concentration and photosynthesis in other plants as well (Soltangheisi et al., 2013; Frydenvang et al., 2015). The decline in photosynthesis rate, transpiration rate, and stomatal conductance with plant aging at the reproductive growth phase is probably due to the phenologically induced reduction in growth rates or the beginning of senescence at the end of the experiment that was demonstrated to occur in other plants as well (Tang et al., 2005).

### Phosphorus Accumulation, Distribution, and Efficiency

Phosphorus concentration increased with the increase in P supply in all plant organs (Figures 5A, 6A). In the leaves, P concentration reached sufficient levels of 4.0–5.5 mg g^–1^ under 60 mg L^–1^ P supply, in accord with previous results for hemp (5–6 mg g^–1^; Iványi and Izsáki, 2009) and for medical cannabis at the vegetative growth stage (3–4 mg g^–1^; Shiponi and Bernstein, 2021). The leaves in P-“hungry” plants (from the 5 and 15 mg L^–1^ treatments) and leaves grown under 30 mg L^–1^ P supply contained only ∼20 and ∼40% of the P stored in leaves grown under sufficient P nutrition of 60 mg L^–1^ P, respectively, in both cultivars. Phosphorous levels in the leaves indicate that 60 mg L^–1^ P is the optimal application sufficient to support the maximum plant uptake potential. Yet, since plant uptake and accumulation potential do not necessarily support optimal plant function, additional parameters were considered, such as plant development and physiology and secondary metabolite production.

Phosphorus accumulation at the reproductive stage was substantially higher in the inflorescence than in all other plant organs, while at the vegetative growth stage, the highest accumulation was found in the roots (Shiponi and Bernstein, 2021). When biomass is taken into account, the inflorescences contained ∼80% of all plant P (Figure 7). An increase in P concentration in the nutrient solution decreased the proportion of P accumulated in the inflorescences on account of an increased proportion of P at the vegetative tissues. Unlike the results obtained by Snapp and Lynch (1996) for beans, retention of P in roots was not apparent at the reproductive stage, and a higher proportion of P compartmentation in the root under low P was not found (Figures 5–7). In line with the results we obtained for cannabis at the vegetative and reproductive stages, a decline in root P concentration at maturity was found by Rose et al. (2008) for canola under high and adequate P. The enhanced translocation to the inflorescences is likely a result of breeding for excelled flower yield biomass. The reduction of the proportion of P content in the vegetative tissues under low P may imply a remobilization of P to the reproductive organs.

Distribution of minerals to plant organs is known to change with plant development, and the massive translocation of minerals to the reproductive organs supports growth of the next generation (Snapp and Lynch, 1996; Veneklaas et al., 2012). At maturity, P concentration is typically lower at the vegetative tissues compared to the grains as was found for numerous plants including lupine (Hocking and Pate, 1978), beans (Snapp and Lynch, 1996), wheat (Rose et al., 2007), canola (Rose et al., 2008), and rice (Rose et al., 2010). At the vegetative stage, root uptake is usually a more important source of P than remobilization between plant tissues. During the reproductive stage, remobilization can become a significant source for support of the new growth (Veneklaas et al., 2012). The results we obtained for cannabis suggest that uptake of P had an important role in P supply to the reproductive tissues since the accumulation of P increased with the increase in P supply. However, at the termination of the exponential growth spurt that the cannabis plant undergoes at the beginning of the reproductive phase, uptake was reduced, and remobilization played a more important role in inflorescence growth. Significant P uptake may occur post-anthesis and was suggested to be genetically related (Rose et al., 2007). Plants re-translocate at least 50% of the P contained in senescing leaves (Aerts, 1996). Remobilization of P was suggested to be part of the senescence process and pod filling in beans (Grabau et al., 1986; Snapp and Lynch, 1996), and in petunia, at the onset of flowering, the concentration of P in the vegetative organs decreased, while in the reproductive organ it gradually increased to a maximum concentration at the senescence stage (Zhang et al., 2012). P distribution in medical cannabis at the end of the cultivated plant life cycle as observed in the current study is in accord with previous knowledge on P accumulation in reproductive organs.

Phosphorus concentration in the inflorescence increased with the increase in P input up to 30 mg L^–1^ P and was not affected by further supply (Figures 5, 6). Taken together with retention in roots under 90 mg L^–1^ P in DQ, the lack of increase in P accumulation in the inflorescences under higher P supply could be an indication of a defense mechanism against P toxicity. Phosphorus homeostasis is achieved by many cellular activities, among which are metabolic processes, translocation between tissues, interaction between ions, and membrane transport (Mimura, 1999). In order to maintain cellular P homeostasis, the plant coordinates between various phosphate transporters (Liu et al., 2016). For example, transporters of the PHT1 family are involved in Pi uptake and remobilization and are controlled by a complex regulation network. They are expressed in the roots for Pi uptake from the growing media and are also detected in various shoot organs such as leaves and flowers (Nussaume et al., 2011). Other phosphate transporters take part in organelle Pi transport, energy metabolism, or stress response (Liu et al., 2016). No information is so far available about P transporters in *C. sativa*, and this information is needed to shed light on P remobilization and translocation in the cannabis plant in light of the high P requirements of the plant. The vacuole functions as a primary compartment for Pi storage and remobilization and buffers Pi concentration in the cytoplasm against fluctuation. Under P deficiency, the Pi pool of the vacuole depletes, and when the Pi storage in the vacuole empties, growth ceases (Bieleski, 1973). Resupply of Pi to Pi-deficient plants results in a rapid flux of Pi into the vacuole. Compartmentation of Pi in the vacuole has an important role in Pi regulation under Pi starvation that prevents toxic levels of Pi in the cytoplasm under excess P (Liu et al., 2016). The tight Pi regulation within the cell may be the reason for the lack of visible toxicity symptoms in the current experiment.

P efficiency in the plant is gained by phosphorus utilization efficiency (PUE) and phosphorus acquisition efficiency (PAE) (Föhse et al., 1988; Wang et al., 2010a; Veneklaas et al., 2012; Wu et al., 2013). PAE is a measure of the ability of the root system to obtain P from the soil, and is an integration of many factors including root morphology and architecture, shoot/root ratio, chemical and biological conditions in the rhizosphere, and the type and density of root Pi transporters (Clarkson and Hanson, 1980; White and Hammond, 2008; George et al., 2012). PUE measures the internal P-use requirement and is commonly calculated as DM production to P concentration in the tissue (Balemi and Schenk, 2009b). PAE is frequently estimated as total P uptake/root biomass (Clarkson and Hanson, 1980). To estimate the external P requirement for optimal yield production by the cannabis plants, we calculated yield efficiency for each treatment as the percentage of yield achieved compared with the maximum yield produced. Based on previous studies, 80% of the maximum yield was defined as the threshold for the optimal P requirement (Föhse et al., 1988; Gourley et al., 1993). Both genotypes had a similar PUE response for the production of total DM and yield (Figures 8A,B). As described by Batten (1992), PUE in wheat was higher under P deficiency. Similar results were obtained for potato (Balemi and Schenk, 2009b), cotton (Wang et al., 2018), and lettuce (Neocleous and Savvas, 2019). PUE_*t*_ under low P supplement was significantly higher for DQ, but PUE_*y*_ was higher for RM (Figures 8A,B). Hence, in RM, utilization of P under deficiency is directed more toward reproductive growth than in DQ.

Phosphorus acquisition efficiency increased with P supply and was higher for DQ compared with RM under adequate P nutrition (30–90 mg L^–1^) (Figure 8C). Although root/shoot ratio was higher for RM, DQ’s PAE was higher, which indicates that, in this variety, parameters other than root/shoot ratio played an important part in P acquisition. Among the required traits for efficient P acquisition by plants are root elongation, increased root/shoot ratio, proliferation of root hairs and lateral roots, root secretion that affects chemical and electrochemical properties in the rhizosphere, and membrane transport properties (White and Hammond, 2008; Wang et al., 2010a). As expected, root/shoot ratio decreased with the increase in P supply in both varieties (Figure 8D). Amplified root/shoot ratio under P scarcity is well known as a defense mechanism to promote P uptake (Bieleski, 1973; Shen et al., 2011; Hawkesford et al., 2012).

Wang et al. (2010a) have suggested that the plant’s P efficiency depends mainly on genetic factors. Genetic variation in P efficiency was found in wheat (Ozturk et al., 2005), potato (Balemi and Schenk, 2009a), cotton (Wang et al., 2018), lettuce (Neocleous and Savvas, 2019), and soybean (Wang et al., 2010a,b). Overall, minor variations in PUE were found between the cannabis varieties tested, and PAE was significantly higher for DQ than RM.

The external requirement of P to achieve 80% of the maximum yield in both varieties was 30 mg L^–1^ (Figure 8E). The data presented here suggest that to achieve maximum yield, a minimum supply of 30 mg L^–1^ P, and an optimum P supply range of 30–90 mg L^–1^ P, are required in both cultivars.

### Interplay Between P Nutrition and the *Cannabis* Plant Ionome

Plants require minerals for growth and development. Macroelements that are present at high concentrations in the plant as well as microelements that are accumulated at considerably lower concentrations are essential for plant function and survival (Kirkby, 2012). Interactions between minerals can affect root uptake and *in planta* translocation. Ion concentration in the root solution may therefore impose a competition between minerals, and a scarcity or an excess of minerals can have a substantial effect on plant development (White, 2012).

Similar to results for the vegetative stage (Shiponi and Bernstein, 2021), N and K concentrations in the leaves, stem, and roots did not show a consistent trend in response to P nutrition between varieties (Figures 5, 6), demonstrating a genetic variability in mineral acquisition, translocation, and accumulation (Clárk, 1983). Some variations in the effect of P on mineral concentrations in the plant organs between the reproductive and the vegetative stages, such as for N and P in leaves (Shiponi and Bernstein, 2021), demonstrate also a developmental stage dependency.

An increase in Mg concentration in leaves up to 30 mg L^–1^ P supply was observed at both stages of development for both genotypes. In Shiponi and Bernstein (2021), we proposed acidification of the rhizosphere as a mechanism to induce Ca and Mg deficiency under P restriction. In support of this notion, leachate pH, in the current study for plants grown under P deficiency (5 mg L^–1^), was lower (pH 4.5) than for plants grown under adequate P nutrition (pH 5.8) (Supplementary Figure 3).

Bioaccumulation of heavy metals in food crops and medicinal plants is a matter of concern worldwide due to their toxic effects on human health (Chizzola et al., 2003). Zn, Mn, Fe, Cu, Mo, and Ni are essential heavy metals that can be absorbed by plants *via* root uptake and accumulate to high concentrations (Ashfaque et al., 2016; Goyal et al., 2020). Variability in the extent of uptake and accumulation of heavy metal nutrients in plants is well documented between and within species, and *C. sativa* (hemp) was recognized as a hyper accumulator for heavy metals and is therefore considered a good candidate for soil phytoremediation (Citterio et al., 2003; Sarma et al., 2020). Hence, to maintain a safe product, data on the bioaccumulation potential of microelements in medical cannabis plant organs is required.

A decline in Mn concentration in the stem with P addition, and a maximum curve in leaves’ Mn (Figures 5F, 6F) were obtain also at the vegetative stage (Shiponi and Bernstein, 2021). Manganese is easily translocated from root to shoot with the transpiration stream in the xylem sap and but it moves poorly in the phloem (Loneragan, 1988; Duèiæ and Polle, 2005). Therefore, remobilization is limited, and Mn accumulates to higher concentrations in mature leaves than in young leaves. The retention of Mn in the root under low P supply in medicinal cannabis may reflect the lower transpiration rate in the 5 and 15 mg L^–1^ P treatments. When Mn levels are adequate, high concentrations of Mn can be stored in the roots and the stem and translocate to the shoot when Mn deficiency conditions develop (Clarkson, 1988; Loneragan, 1988). Generally, Mn accumulates in the root under sufficient Mn level (Loneragan, 1988), although a higher shoot/root ratio of Mn concentration is also common (Xue et al., 2004; Farzadfar et al., 2017). Baker (1981) discussed two strategies of plant response to tolerate metal toxicity: accumulators and excluders. Accumulators usually transport the metals to the aerial parts, while the excluders’ response involves maintaining a low concentration in the shoot. Polechońska et al. (2013) found that the bioaccumulator plant, *Polygonum aviculare*, accumulates essential micronutrients (Zn, Mn, and Cu) in the aerial parts, unlike unessential elements that are accumulated in the root (Cd, Ni, and Pb). Xue et al. (2004) documented that in the hyperaccumulator plant *Phytolacca acinosa*, 87–95% of the Mn was translocated to the shoot. In medicinal cannabis, we found that at the vegetative-stage Mn was retained in the root, whereas at the reproductive stage the highest concentrations in the shoot were found under adequate P nutrition. *Cannabis* is known as a good bioaccumulator; thus, accumulation in the shoot is not surprising. The plant strategy can be transformed from excluder to accumulator during the plant life cycle (Baker, 1981), and it may be the reason for the differences between Mn accumulation in the plant organs at different development stages.

We identified a decrease in Zn concentrations in the root with the increase in P concentration in both the vegetative and the reproductive phase for both cultivars, and the P × Zn interaction was discussed in detail in Shiponi and Bernstein (2021). Inflorescence Zn concentration was ∼40% higher in DQ compared with RM, demonstrating a genetic variability. Genetic variability was reported before to affect heavy metal accumulation in plants (Baker and Brooks, 1989; Malik et al., 2010), and in cannabis, it can be explored for reduction of heavy metal accumulation in the pharmaceutical product.

The safety of medicinal cannabis consumption and the safe limit of heavy metal concentration in the product are not yet well researched and are topics of interest in light of the growing global demand. Thus, there is an increasing necessity for the regulation and restriction of heavy metal concentrations in medicinal cannabis (Gauvin et al., 2018; Nie et al., 2019). Further research on the effect of the plant genotype and environmental factors in relation to heavy metal acquisition is necessary.

### Cannabinoids

Cannabis is one of the oldest plant sources for medicine. It is known for centuries for its medicinal potential and has been used traditionally for thousands of years for the treatment of a wide array of ailments (Gonçalves et al., 2019). Recent changes in regulations have allowed proliferation of medical studies, and significant progress has been made toward the understanding of the potential of the plant-produced cannabinoids, and their interactions with other biologically active secondary metabolites in the plant, for modern medicine (Citti et al., 2018). Filling the medical knowledge gap, as well as knowledge concerning the influence of agro-technologies on the concentrations and ratios between the pharmacological compounds, is of high priority for optimizing the medicinal value of the product.

The production of secondary metabolites is known to be affected by environmental factors (Verpoorte et al., 2002; Ramakrishna and Ravishankar, 2011; Gorelick and Bernstein, 2014). Among other secondary metabolites, cannabis plants produce cannabinoids that are biosynthesized and stored in trichomes located mainly on the plant inflorescence. Cannabis plants differ in their cannabinoid contents due to genetic and environmental factors (Chandra et al., 2017; Danziger and Bernstein, 2021a). Abiotic stressors were found to induce changes in the cannabinoid profile in the cannabis plant (Flores-Sanches and Verpoorte, 2008; Backer et al., 2019; Caplan et al., 2019; Jalali et al., 2019; Gülck and Møller, 2020; Yep et al., 2020; Saloner and Bernstein, 2021), and in the current study, P nutrition was found to elicit changes in cannabinoid concentrations in the two genotypes tested. We found a reduction in concentrations of most tested cannabinoids with an increase in P application (Figure 9), which negatively correlated with yield production (Figure 11). Although the concentrations of many cannabinoids (especially THCA and CBDA) reduced, the amount of cannabinoids produced per plant increased with P, suggesting that the optimal P application is 30 mg L^–1^ in DQ and 30–90 mg L^–1^ in RM (Figure 10).

Previous studies on P effect on secondary metabolites and essential oil production found a variety of responses to increased P application. P increased essential oil production in *Cymbopogon nardus* (Ranaweera and Thilakaratne, 1992; Ranaweera et al., 1992), *Olearia phlogopappa* (Dragar and Menary, 1995), cannabis (Coffman and Gentner, 1977), dragonhead (Said-Al Ahl and Abdou, 2009), and basil (Ichimura et al., 1995). Essential oil content was not affected by P in sage (Rioba et al., 2015) and basil (Ichimura et al., 1995) and by a moderate P application in cannabis (Bernstein et al., 2019b) and was reduced in *Olearia phlogopappa* (Dragar and Menary, 1995). An increase in THCA and CBDA concentrations as a response to P deficiency are unlikely to be a direct effect of P on the biochemical pathways due to the crucial role of P in cannabinoid biosynthesis (Fellermeier et al., 2001; Flores-Sanches and Verpoorte, 2008; Sarma et al., 2020). The negative correlation between THCA and CBDA, and inflorescence yield production (Figure 11) indicates that a dilution effect may be a possible mechanism for the reduction in their concentration.

When Pearson correlation coefficient was performed on THCA, CBDA, and total P in the plant, the correlation was weaker compared to yield production (*R*^2^ = RM: 0.59, 0.37 and DQ: 0.32, 0.31 for THCA and CBDA, respectively). The lower correlation with P compared with inflorescence yield suggests that the influence on the cannabinoid concentrations is probably due to yield increase and not a direct effect of P. Caplan et al. (2017) found, that in cannabis, NPK application reduced THCA, THC, and CBGA concentration and increased yield. Corresponding to our results, a negative correlation between THCA and inflorescence yield was found, and the total cannabinoids per plant increased with NPK. In our study, both genotypes analyzed responded similarly to the P treatments, and only minor changes were observed, this is despite the very different chemovars (a high THC vs. a CBD/THC balanced profile). Unlike DQ that did not show a response to P addition above 30 mg L^–1^ in total cannabinoid production, RM responded with a slight increase that might indicate a potential for yield increase with P addition for certain genotypes, which should be tested further. The reduction in THCA concentration in response to P addition was slightly higher for RM compared with DQ; THCVA was reduced with P in RM and was only slightly affected in DQ.

These results demonstrate that the effect of P nutrition on the cannabinoid profile may be genotype specific, and genetic differences should be explored for the optimization of the secondary metabolite profile by P-nutrition technologies. More research is needed on medicinal effects of cannabinoids and their interactions, in order to direct growing techniques for production of medical product with a desirable cannabinoid profile.

### In Summary

Phosphorus nutrition considerably affects morpho-physiology of medicinal cannabis and its chemical profile. No signs of P toxicity were found under the concentration range tested; however, at the two lowest P supply rates (5 and 15 mg L^–1^ P), P deficiency reduced plant growth and physiological function, induced leaf chlorosis, but increased root/shoot ratio and PUE. Moreover, 80% yield efficiency was achieved under 30 mg L^–1^ P supply in both genotypes but was higher for DQ. Although both genotypes responded similarly to the P application dosages, DQ’s yield production was not affected by P addition above 30 mg L^–1^, unlike RM that responded with a small increase to P addition up to 90 mg L^–1^ P. Thereby, DQ demonstrated best performance under lower P application compared with RM that slightly increased in yield under high P supply. Taken together, our results demonstrate that the optimal P nutrition needs to be adjusted to the target product. The lowest recommended P supply for optimal yield quantity is 30 mg L^–1^ P; under higher concentrations up to 90 mg L^–1^ P, yield quantity remains optimal; and P deficiency stress (5–15 mg L^–1^ P) can be used to stimulate higher concentrations of the major cannabinoids. More research needs to be conducted on specific genotypic responses to P addition above the optimal dosage.

## Data Availability Statement

The original contributions presented in the study are included in the article/Supplementary Material, further inquiries can be directed to the corresponding author/s.

## Author Contributions

NB planned the experiments. SS carried out the experiments. NB and SS wrote the manuscript. Both authors contributed to the article and approved the submitted version.

## Conflict of Interest

The authors declare that the research was conducted in the absence of any commercial or financial relationships that could be construed as a potential conflict of interest.
