# Supplementary material for: The Highs and Lows of P Supply in Medical Cannabis: Effects on Cannabinoids, the Ionome, and Morpho-Physiology
Source: Front Plant Sci. 2021 Jul 15;12:657323. doi: 10.3389/fpls.2021.657323 (PMC8320666; doi:10.3389/fpls.2021.657323)
Supplement: Supplementary file 1 [file Data_Sheet_1.pdf]

The highs and lows of P supply in medical cannabis: effects on the cannabidiome, ionome and morpho-physiology

Sivan Shiponi, Nirit Bernstein*

* Institute of Soil water and Environmental Sciences, Volcani Center, Israel, Nirit@agri.gov.il

Supplemental Material

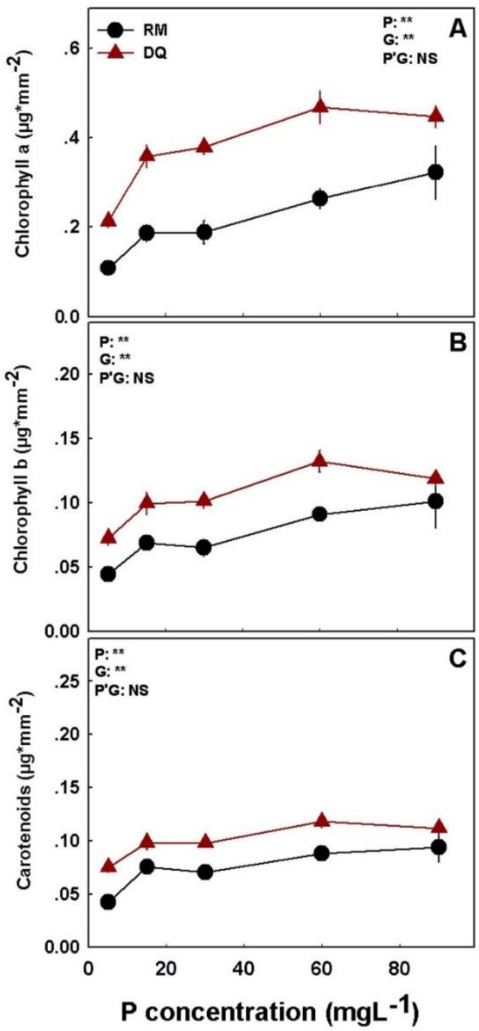

<u>Fig. 1. Supplemental</u>: Effect of P application on the concentration of photosynthetic pigments in two medical cannabis cultivars, RM and DQ. Chlorophyll a (A), chlorophyll b (B) and carotenoids (C). Presented data are averages ±SD (n=6). Results of two-way ANOVA indicated as ** *P* <0.05, *F*-test; NS, not significant *P* >0.05, *F*-test. In the ANOVA results P*G represents the interaction between P and genotype.

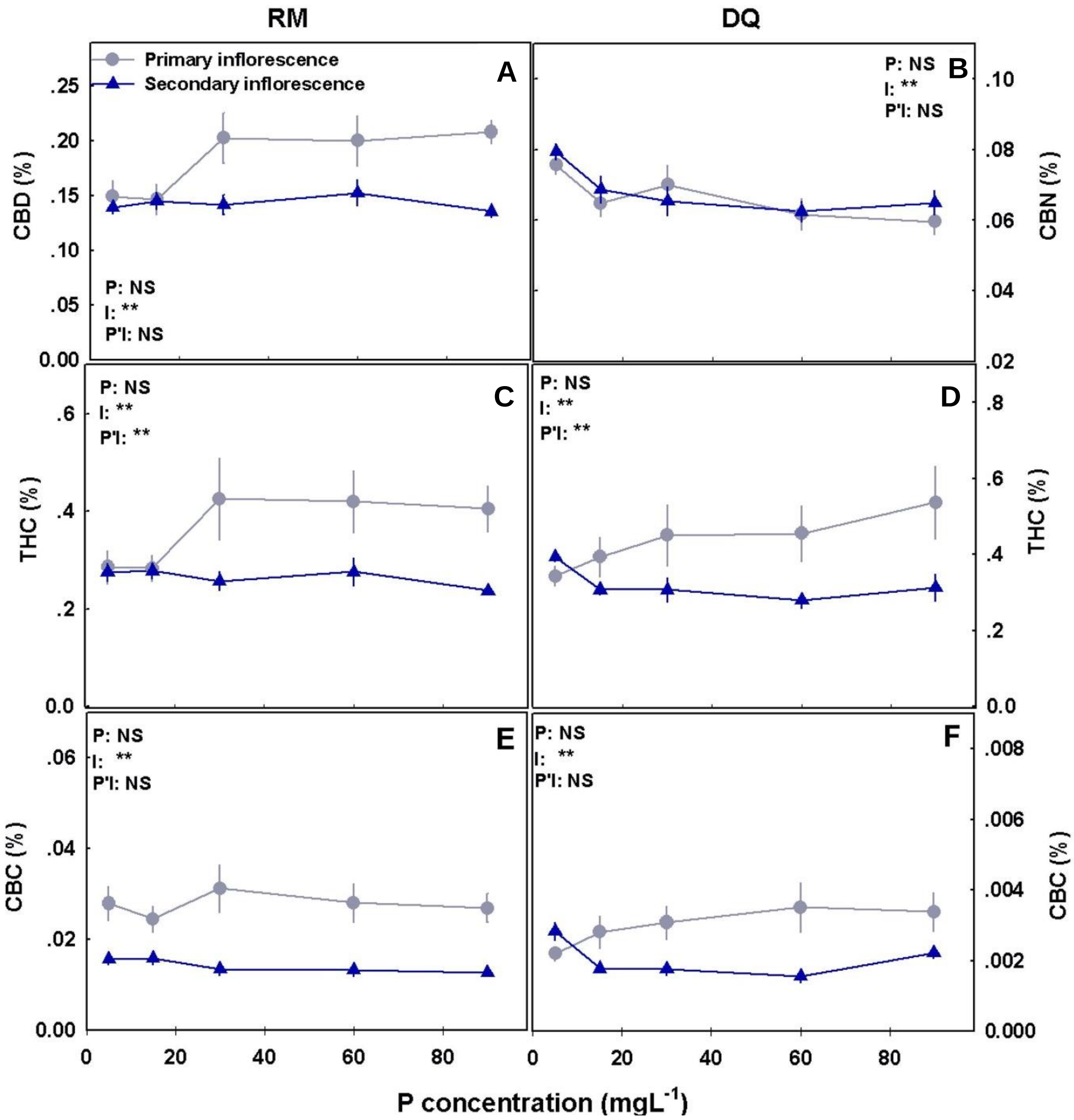

<u>Fig. 2. Supplemental</u>: Effect of P application on cannabinoid concentration**s** in primary and secondary apical inflorescences of medical cannabis plants, from 2 cultivars, RM and DQ. CBD (A), CBN (B), THC (C,D), CBC (E,F), Presented data are averages ±SD (n=6). Results of two-way ANOVA indicated as ** P <0.05, F-test; NS, not significant P >0.05, F-test. In the ANOVA results P*I represents the interaction between P and inflorescence location.

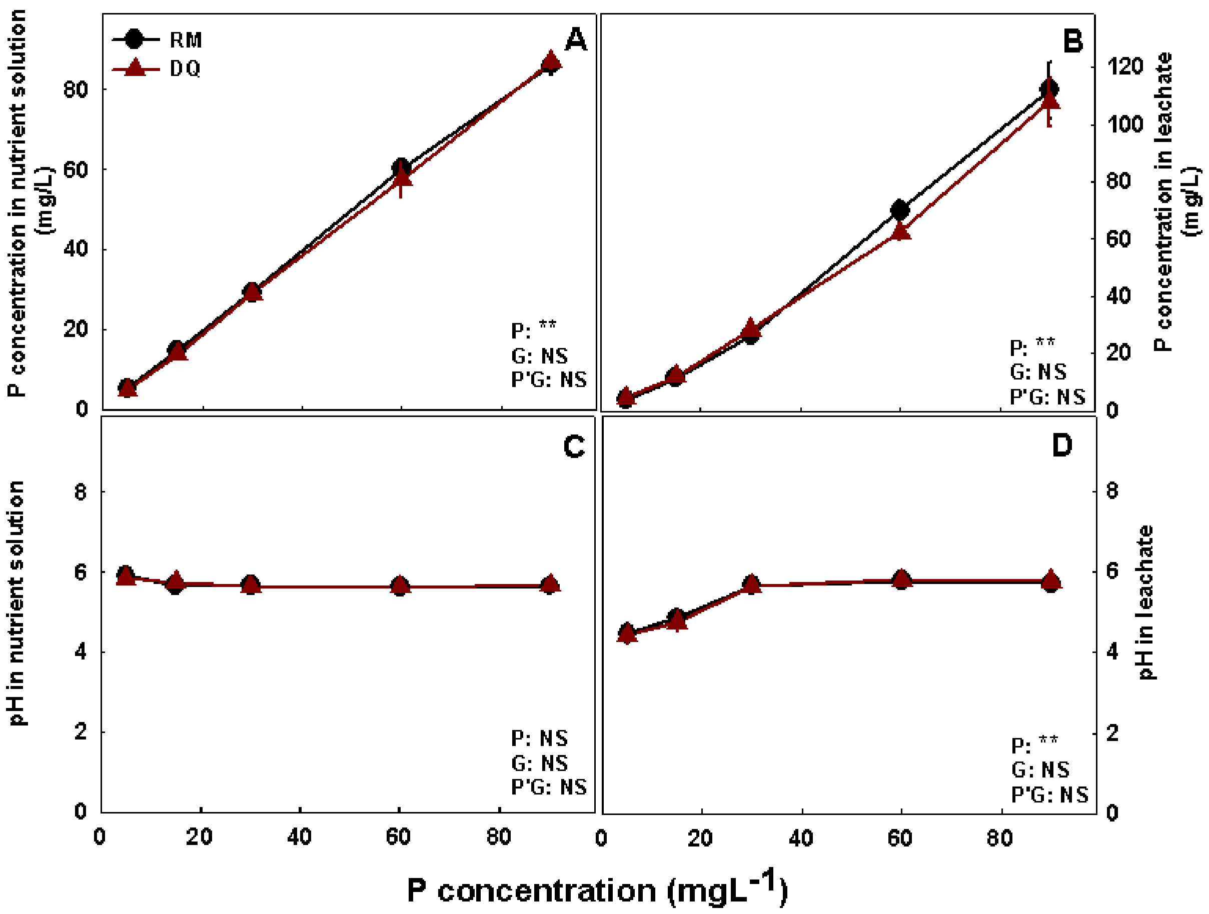

<u>Fig. 3. Supplemental</u>: P concentration in the irrigation solution and the leachate (A,B) and pH in the irrigation solution and the leachate (C,D) throughout the experiment duration, from two genotypes, RM and DQ. Presented data are averages ±SD (n=8). Results of two-way ANOVA indicated as ** $P<0.05$, F-test; NS, not significant $P>0.05$, F-test. In the ANOVA results P*G represents the interaction between P and genotype.
